# *In silico* identification of AMPylating enzymes and study of their divergent evolution

**DOI:** 10.1038/srep10804

**Published:** 2015-06-03

**Authors:** Shradha Khater, Debasisa Mohanty

**Affiliations:** 1 Bioinformatics Center, National Institute of Immunology, Aruna Asaf Ali Marg, New Delhi – 110067, India

## Abstract

AMPylation is a novel post-translational modification (PTM) involving covalent attachment of an AMP moiety to threonine/tyrosine side chains of a protein. AMPylating enzymes belonging to three different families, namely Fic/Doc, GS-ATase and DrrA have been experimentally characterized. Involvement of these novel enzymes in a myriad of biological processes makes them interesting candidates for genome-wide search. We have used SVM and HMM to develop a computational protocol for identification of AMPylation domains and their classification into various functional subfamilies catalyzing AMPylation, deAMPylation, phosphorylation and phosphocholine transfer. Our analysis has not only identified novel PTM catalyzing enzymes among unannotated proteins, but has also revealed how this novel enzyme family has evolved to generate functional diversity by subtle changes in sequence/structures of the proteins. Phylogenetic analysis of Fic/Doc has revealed three new isofunctional subfamilies, thus adding to their functional divergence. Also, frequent occurrence of Fic/Doc proteins on highly mobile and unstable genomic islands indicated their evolution *via* extensive horizontal gene transfers. On the other hand phylogenetic analyses indicate lateral evolution of GS-ATase family and an early duplication event responsible for AMPylation and deAMPylation activity of GS-ATase. Our analysis also reveals molecular basis of substrate specificity of DrrA proteins.

AMPylation or adenylylation is a post-translational modification (PTM) involving the covalent attachment of AMP moiety onto the side chains of threonine/tyrosine residues of proteins^1^. Recent proteomics studies suggest that AMPylation of proteins is more ubiquitous than is generally acknowledged. Experimental studies have demonstrated that AMPylation is involved in wide array of biological processes, ranging from pathogenesis in several animal hosts to regulation of nitrogen metabolism in bacteria and regulation of signaling pathways in eukaryotes^1^,^2^,^3^,^4^. It has been shown that AMPylation is catalyzed by three different families of enzymes, namely, Fic (Filamentation induced by cAMP), DrrA and GS-ATase (Glutamine Synthetase Adenylyltransferase)^2^,^5^,^6^. Among these AMPylating enzyme families, Fic, the largest of the three contains approximately 7000 proteins from all three kingdoms of life^7^. Most of the Fic protein associated with host pathogen interactions AMPylate switch I region of RhoGTPase, rendering them inactive and hence causing collapse of actin cytoskeleton^6^,^8^,^9^,^10^. Recent discoveries have revealed wide array of non-GTPase Fic substrates^11^,^12^,^4^. Fic domain containing proteins are defined by the presence of a C-terminal motif, HxFx[D/E]GN[G/K]R, containing an invariant histidine residue. Outside this conserved motif Fic domains share little sequence similarity, though their overall fold is conserved. The vast sequence divergence in Fic family is reflected in diverse post translational modifications that Fic domains can catalyze. Apart from catalyzing transfer of moieties like GMP and UMP, structurally similar to AMP^13^, certain Fic domains can also catalyze phosphocholine transfer^14^ and phosphorylation^15^,^16^ reactions.

While catalytic activity of Fic domains is a more recent finding, the enzymatic ability of GS-ATase was discovered almost four decades ago^2^,^17^,^18^. GS-ATase regulates Glutamine Synthetase (GS), a key enzyme in nitrogen metabolism of enteric bacteria. The C-terminal Adenylyl Transferase (AT) domain AMPylates GS, thereby inhibiting it whereas, the N-terminal Adenylyl Removase (AR) domain deAMPylates converting it back to the active form (Fig. 1). The functionally antagonistic domains share 24% sequence identity, mostly restricted to the active site region^19^, and have similar structural fold (supplementary Figure S1); indicative of evolution from a common ancestor and divergence through gene duplication^20^. Interestingly, recently characterized AMPylating domain in *Legionella pneumophila* effector protein, DrrA^5^ also adopts a structural fold similar to GS-ATase. N terminal domain (ATase) of DrrA (Fig. 1) AMPylates hosts Rab1b on switch II region, thereby impeding its inactivation. This helps in hijacking the vesicular transport and hence aids in *Legionella*’s survival in lysosomal vacuoles^21^. Even though DrrA and GS-ATase share a common structural fold and most catalytic residues are conserved, they have distinctly different substrate specificities.

Fic domain proteins do not share any sequence or structural similarity to GS-ATase but shares sequence similarity with another protein family known as Doc (Death on curing). PFAM^22^ classifies Fic domains together with Doc. Doc forms the toxin part of toxin-antitoxin module of *E.coli* phage P1. These proteins contain a degenerate Fic motif, HPFx(D/E)GN(G/K)R, with the His being essential for its catalytic function^6^,^9^. Recent studies have shown that Doc domains catalyze phosphorylation^15^,^16^ reaction similar to kinases. Another type III effector protein, avirulence protein B (AvrB) from *Pseudomonas syringae* shares structural similarity with Fic/Doc family of proteins. AvrB causes hypersensitive death of plant cells by targeting host immune resistance protein Rin4^23^. Though AvrB lacks the conserved motif, it contains all the alpha helices and β hairpin (missing in doc) of Fic domain and have similar peptide binding mode too^10^,^23^. In view of the sequence and structural similarity between Fic, Doc and AvrB, they have been grouped together as a single superfamily called Fido (Supplementary Figure S1); though they do not catalyze same reaction.

It is necessary to understand how these enzyme families have evolved across various organisms to generate functional diversity by subtle changes in sequence and structures of the proteins. Evolutionary analysis of functional diversity across organisms requires *a priori* identification of AMPylation domains and their classification into various subfamilies. Even though AMPylation is involved in diverse biological processes, only a handful of AMPylating enzymes have been experimentally characterized. Due to high sequence divergence within each family of AMPylating enzymes all members cannot be identified by a simple BLAST search or profile based tools like PSI-BLAST. They are also limited in their ability to distinguish non-AMPylating from the AMPylating members. Therefore, in this work we have attempted to use machine learning approach like SVM and profile based approach like HMM to develop and benchmark a computational protocol for identification and classification of AMPylation domains. Using this computational approach we have identified large number of AMPylation domains belonging to various subfamilies in all three kingdoms of life. Phylogenetic analysis has also identified putative isofunctional subfamilies expanding the functional diversity of Fic domains. Analysis of phylogeny and synteny of these AMPylation domains suggested evolution of AMPylating enzymes, specifically Fic domains by extensive horizontal gene transfer. This was confirmed by the presence of many Fic proteins on highly unstable Genomic Islands. In addition, we have also attempted to decipher sequence determinants of the substrate specificity of DrrA type AMPylation domains towards a variety of Rab proteins.

## Results

### Classification of Fic, Doc and AvrB proteins

Extensive sequence diversity coupled with functional divergence of Fic/Doc family poses a major challenge for *in silico* identification of AMPylation domains and their classification into various subfamilies like Fic, Doc and AvrB. Even though iterative profile based searches increases the repertoire of Fic/Doc proteins compared to pair-wise BLAST search, it is often found that such methods cannot distinguish between the subfamilies. We wanted to investigate if machine learning based SVM classifiers and sensitive profile based HMMs can be used for identification and classification of AMPylation domains.

Machine learning approaches like SVM (Support Vector Machines)^24^ have the ability to derive hidden patterns from complex datasets^25^,^26^. We have used standalone SVMs where single feature like amino acid composition (AAC) or dipeptide composition (DPC) is used to encode the sequence information and also hybrid SVMs where a combination of features is used. Training and validation of SVM models were carried out as mentioned in methods section. Supplementary Table 1 and Fig. 2 summarize the results of 5-fold cross validation test for various SVM classifiers analyzed in the current work. The bar graphs in Fig. 2 summarizes the statistical parameters obtained from 5-fold cross validation test averaged over Fic, Doc and AvrB classifications, while Supplementary Table 1 gives the details of the validation with C and F1 values as additional measures of performance. The MCC values indicate that out of the different standalone SVM classifiers which use single features, tetrapeptide composition has significantly better performance. However, the hybrid SVMs which combine dipeptide composition with AAC have performance comparable to standalone SVM with tetrapeptide composition and best performance is achieved when feature vectors are obtained by combining AAC, dipeptide and tetrapeptide composition. The value of parameter “C” (trade-off value) at which these results were obtained was 0.01 for former and 0.001 for later. Principal Component Analysis (PCA) of feature vectors was also carried out on complete datasets of standalone SVM classifiers (supplementary Figure S2). PCA analysis clearly shows that segregation and clustering of the three sub-families is much better when tetrapeptide composition model is used, revealing a trend similar to that obtained from calculation of MCC and percent S values. Since SVM analysis of other protein families has shown dipeptide composition to be adequate for classification of various functional properties, it was intriguing why classification of AMPylation domains requires tetrapeptide composition. Interestingly, analysis of tetrapeptide composition revealed that out of the ten 4-mers which are major sequence determinants of Fic, Doc and AvrB domains, 5 tetrapeptides were part of the Fic/Doc conserved motif. As subtle variations in the conserved motif distinguish these sub families, the SVM based on tetrapeptide or their hybrids were able to capture this information and hence performed better than other SVM classifiers.

**Figure 1:**
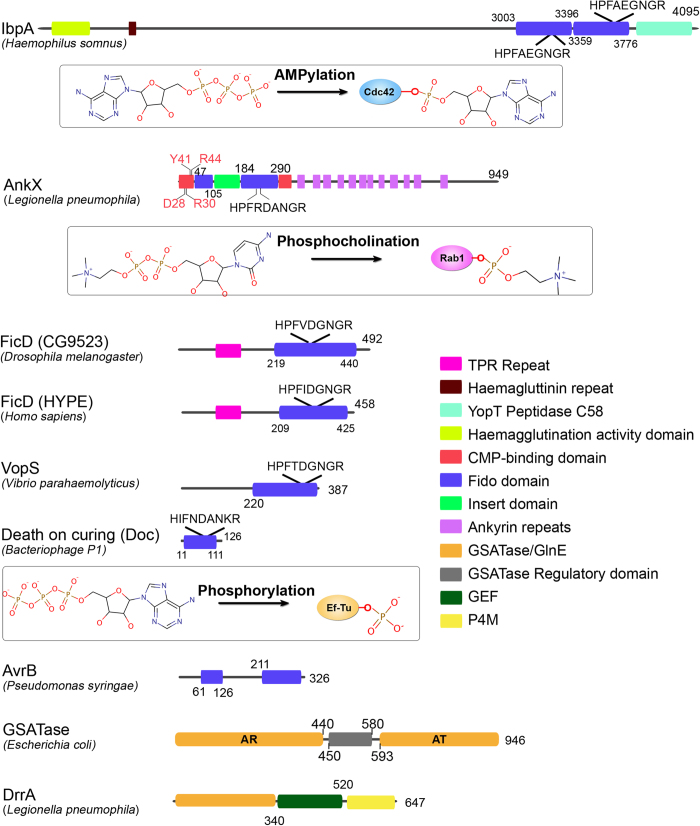
Organization of Fido and GS-ATase domains. Fido and GS-ATase domains co-occur with a number of different functional domains. Insets show the PTMs catalyzed by these enzymes.

**Figure 2:**
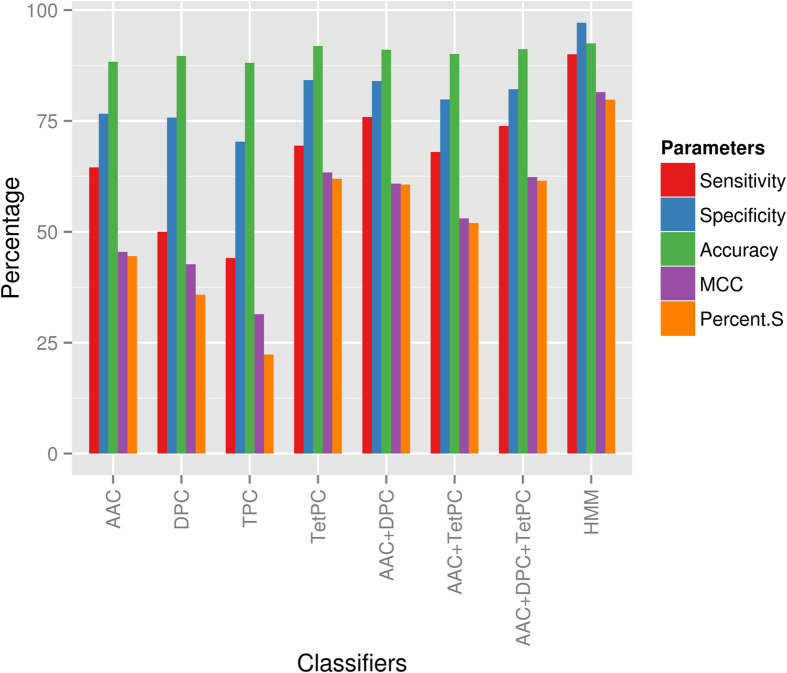
Performance of SVM and HMM classifiers. Performance of various classifiers for distinguishing between Fic, Doc and AvrB family of enzymes. Seven SVM classifiers and HMMs were evaluated using fivefold cross validation technique. Different statistical parameters averaged over all three families have been plotted for each classifier, while values for individual families are given in Supplementary Table 1.

Hidden Markov Models (HMM), a fast and sensitive profile based method has been widely used for classification and identification of different protein domains^27^,^28^,^29^. Use of HMMs to classify the sub-families showed marked improvement in the statistical parameters. Performance of HMMs was also evaluated using a fivefold cross validation technique. MCC and percent S values (Supplementary Table 1 and Fig. 2) showed that HMM outperformed both standalone and hybrid SVM models. The performance of AvrB HMM model was exceptionally good due to no significant sequence similarity between AvrB and Fic/Doc family. The superior performance of HMM, than SVM, could be due to presence of additional helices and huge insertions in Fido family members (Fig. 1 and supplementary Figure S1). The profile based method is also better suited to overcome insertions and deletions than SVMs.

Since the HMM method was found to be superior to SVM method in our benchmarking study for identification and classification of Fic, Doc and AvrB families, for analysis of AnkX, AR, AT and DrrA family members only HMM profiles were developed. Hence, only the developed HMMs for various subfamilies were used to identify AMPylating domains from nr database (Table 1). Similarly for subsequent phylogenetic analysis of Fic, Doc, AR, AT and DrrA families sequences were identified from various genomes by using HHM profiles only.

### Classification and genomic search for AnkX like proteins

AnkX from *Legionella pneumophila* is a multi-domain protein containing Fic domain, a CMP-binding domain and several ankyrin repeats (Fig. 1). The Fic domain of AnkX catalyzes transfer of phosphocholine group from CDP-choline onto Rab1^14^. Since the Fic domain of AnkX shares a high sequence similarity with other Fic domains, BLAST search using Fic domain of AnkX as query gives a number of Fic domains which catalyze AMPylation reaction. On the other hand, no separate HMM profile could be built for AnkX like domains because the number of experimentally characterized AnkX type proteins or their close homologs was very few in number. However, experimental studies have revealed that, CMP-binding domain of AnkX harbors important active site residues which is necessary for binding of CDP-phosphocholine to AnkX^30^. Interestingly, BLAST search for CMP binding domains of AnkX always yielded proteins containing Fic domains along with CMP binding domains, thus suggesting that such CMP-binding domains are exclusively present in AnkX like proteins. Therefore, a HMM profile for the CMP binding domain of AnkX was built and this profile was used to search remote homologs of AnkX. A slightly higher e-value cut-off was used to include remotely related sequences. This increased the probability of occurrence of false positive hits in our search. These false positives were eliminated by choosing only those hits which contained a Fic domain and had substrate binding residues of CMP-binding domain conserved. This protocol helped us to identify 4 new AnkX type sequences which could not be identified using BLAST search. This computational protocol also identified an AnkX type protein (NCBI GI: 496438677) which has been wrongly annotated as UDP-N-acetylmuramyl pentapeptide synthase.

### Phylogenetic analysis reveals isofunctional subfamilies

**Figure 3:**
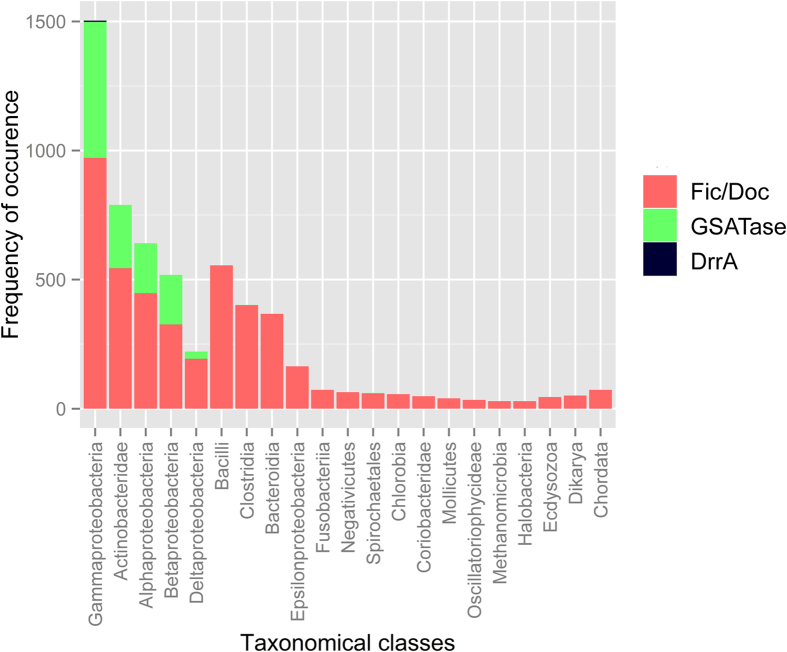
Taxonomic distribution of AMPylators from three different families. Taxonomic distribution of Fic, GS-ATase and DrrA type AMPylation domains identified in nr database using profile HMMs developed in the current study. The bars represent number of AMPylation domains in different taxonomical classes. Distribution of Fic/Doc family is represented in red color, GS-ATase in green and DrrA in black.

**Figure 4:**
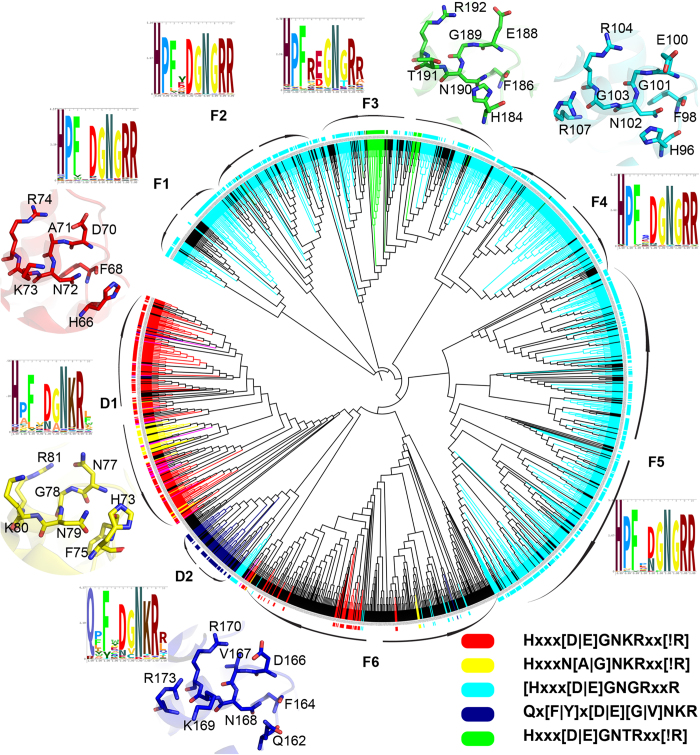
Conserved motif of each clade. Fic/Doc sequences were manually clustered based on their motifs. Consensus motifs of each of the 5 clusters obtained were mapped on to the Fic/Doc phylogenetic tree using various colors. Active sites of each of the 5 clusters have been mapped on available 3D structures or modeled structures. The legend represents the consensus motif. HMM logo for Fic motif of each clade has also been represented here. As clade F6 did not have a conserved Fic motif the HMM logo was not represented.

Analysis of Fic and Doc domains identified by our HMM search in nr database revealed that, though Fic/Doc sequences were majorly from bacterial classes smaller number of proteins are also present in other two kingdoms of life i.e. Archaea and eukaryotes (Fig. 3). Based on phylogenetic analysis earlier reports had classified Fic/Doc family into three distinct subfamilies^31^,^32^. To analyze the evolution of these proteins in greater details 1883 representative sequences were used to reconstruct the evolutionary history of Fic/Doc family. An analysis with larger number of sequences produced a slightly different result compared to Anantharaman *et al.* 32. As reported earlier, Doc-like proteins (colored in red) are segregated from other Fic proteins and cluster as separate clade (Fig. 4). The Doc clade can be further divided into sub-clades D1 and D2. Other Fic proteins cluster into six different clades (F1-F6). To understand if the clustering has a functional significance the catalytically important Fic motif of all the sequences were extracted. Most of the Fic/Doc sequences could be clustered into five groups based on their Fic motif. The consensus motifs are: Hxxx[D|E]GNKRxx[!R], HxxxN[A|G]NKRxx[!R], Hxxx[D|E]GNGRxxR, Hxxx[D|E]GNTRxx[!R], Qx[F|Y]x[D|E][G|V]NKR. These motifs were mapped onto the phylogenetic tree using different colors and interestingly the partitioning based in these motifs is largely in agreement with the phylogenetic tree (Fig. 4).

Detailed structure based analyses were also carried out to locate the conserved sequence motifs for Fic/Doc families on three dimensional structure of Fido domains. The consensus motifs from representative members of the five groups were either mapped on available crystal structures or modeled structures. Supplementary Figure S3 shows the three dimensional structure of the sequence stretch corresponding to the conserved sequence motif (shown in Fig. 4) in HpFic from *Helicobacter pylori* (PDB ID: 2F6S). As can be seen this sequence stretch HPFLEGNGRATR corresponding to the residues 96-107 in 2F6S adopts a helix-turn-helix (α4-turn-α5) structure which harbors most the active site residues (shown in bold in supplementary Figure S3) of Fic domain. Interestingly superposition of available crystal structures of Fic domains indicate this region to be structurally conserved, even though there are subtle variations in the amino acid sequence. Since this sequence stretch constitute a part of the active site pocket of the Fic domain subtle changes in the conserved sequence motif in various subfamilies of Fic domains can potentially alter their substrate specificities. Figure 4 also shows the conformation of the conserved motif based on crystal structures or homology models from different Fic/Doc families. As can be seen despite variations in the sequence motifs in different subfamilies, the backbone conformation of the sequence stretch and orientations of His, Asp and Arg residues remain conserved across subfamilies, thus further supporting their key role in function.

The results from our sequence and structure based analysis indicated that the sequences clustering together in the phylogenetic tree (Fig. 4) might represent isofunctional subfamilies. Most of the Fic proteins (F1 – F5) cluster together under the motif Hxxx[D|E]GNGRxxR and Doc proteins (D1) under Hxxx[D|E]GNKRxx[!R]. This active site based partitioning is not perfect and we observed some anomalies. A small number of Fic proteins (colored in green) from clade F3 has slightly different motif where instead of second glycine a threonine is present and the last arginine is absent. Last arginine in the conserved motif is involved in binding the γ phosphate of ATP and orientation of α phosphate for the incoming nucleophilic attack^33^,^34^. Interestingly our structure based analysis using molecular dynamics simulations (Khater S and Mohanty D, unpublished work) revealed that this group of proteins contains a modified inhibitory helix involved in regulation of AMPylation activity, as demonstrated by Engel *et al.* in case of VbhT^33^. Another small group of proteins within Doc clade D1 (colored yellow) has the catalytically critical aspartate residue mutated to aspargine. The aspartate residue is involved in metal ion binding which in turn binds α and β phosphate of ATP. It is possible that these Fic and Doc domains (in clades F3 and D1) which lack crucial conserved residues might be catalytically inactive. However, the known functional diversification in Fic enzymes and the selection pressure to retain these variations in diverse organisms, points at functional importance of these variations. In fact subtle changes in Fic motif has been seen to be associated with different molecular functions^31^. Hence, these proteins might be inactive for catalyzing AMPylation reaction, but still have other biological function or have different substrate specificity or they might be utilizing alternate binding mode for substrate recognition.

Another drastic substitution is seen in the clade D2 of Doc subfamily where the catalytically essential Histidine is replaced by Glutamine. This clade contains 45 sequences having a consensus motif of Qx[F|Y]x[D|E][G|V]NKR. Like other Doc proteins D2 proteins are usually single-domain proteins. The taxonomical distribution of these proteins is restricted to bacterial classes. In IbpA, a His to Ala mutation could not abolish AMPylation activity completely^35^ and residual activity has been reported in pseudokinases which have substitution in their catalytic aspartate^36^. Also, significant residual activity was also seen in H to Q mutants of human liver alcohol dehydrogenase^37^. The selection pressure to retain D2 clade and the above mentioned literature evidences support the hypothesis that these proteins might not be catalytically dead; instead they might have diverse molecular or biological functions. The logos of active site of all the members from same clade showed better conservation than outside. Therefore, phylogenetic analysis of Fic/Doc family reveals that the full range of functional divergence of Fic family might still be unexplored by experimental studies. Our analysis shows that though majority of Fic domains are AMPylators, at least three distinct groups of proteins might have other functions which are yet to be identified. These novel Fic domains identified by our genome wide search and phylogenetic analysis could be interesting candidates for experimental studies.

### Extensive evidence of Horizontal Gene Transfer in Fic/Doc family

**Figure 5:**
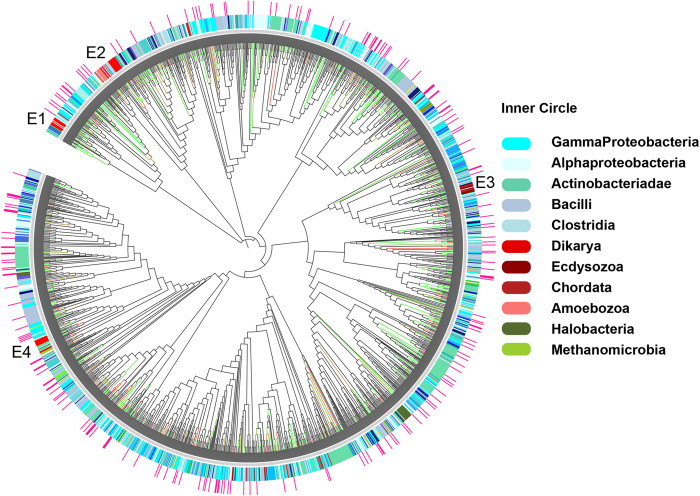
Phylogenetic tree reveals extensive HGT in Fic/Doc family. Taxonomical distribution of the proteins has been mapped onto the phylogenetic tree by labeling the inner circle surrounding the phylogenetic tree in different color based on source organism of the corresponding sequence. A pink line is shown on the outer circle if the corresponding Fic/Doc gene is located in a genomic island as predicted by IslandViewer 43,44. Location of large number of leaves of the phylogenetic tree on genomic islands indicates extensive horizontal gene transfer in Fic/Doc family. The branches have been colored based on bootstrap values. Green indicates high bootstrap value whereas red indicate low bootstrap value and hence low confidence.

Another remarkable feature of the Fic/Doc phylogenetic tree (Bacteria – Blue shades, Archaea – Green shades and Eukaryotes – Red shades) was that all groups are polyphyletic i.e. species of bacteria, archaea and eukaryotes are mixed in all the branches (Fig. 5). Hence, the tree is not in congruence with the canonical tree of life, indicating extensive horizontal gene transfers (HGT) have occurred during the evolution of Fic/Doc proteins. Its extensive occurrence in bacteria, sporadic presence in eukaryotes and archaea combined with absence of archaeo-eukaryotic branches suggest that Fic/Doc domain might have evolved in bacteria and subsequently spread laterally into the other two kingdoms. The phylogenetic tree shows the presence of four major eukaryotic groups (marked as E1 – E4) that do not cluster together. Also, further analysis revealed differences in their conserved Fic motif. Hence, eukaryotic Fic/Doc domains seem to have evolved through multiple horizontal gene transfers (HGT) (details of which is discussed in supplementary text in Supplementary File 1). The substantial amount of HGT events in Fic domains impelled us to look for further evidences and to search for the mode of genetic transfer.

**Figure 6:**
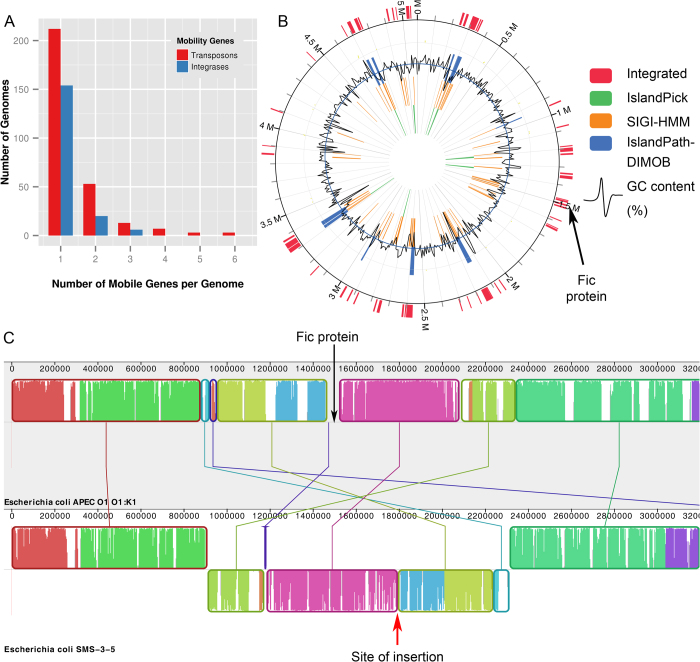
Occurrence of Fic/Doc proteins on genomic islands. (**A**) Bar plot depicts number of genomes containing different number of mobility genes in the neighborhood of Fic/Doc domain containing genes. (**B**) Circular map of *Escherichia coli APEC O1* chromosome depicting genomic island as predicted by IslandViewer. The lines on the inner circles are colored based on the tools used for prediction. The wavy black line indicates variation of %GC content. The pink lines on the outer circle indicate location of genomic island as predicted by IslandViewer 43,44. Fic protein of *E. coli APEC O1* (Nucleotide position: 1480490 to 1480975) is marked by an arrow and it lays within region of predicted genomic island. (**C**) Genome alignment of Fic containing *E. coli APEC O1* and *E. coli SMS-3-5* (lacks Fic) using progressive Mauve 63,64. Similar colored blocks in two genomes connected by lines represent homologous regions. The genomic regions in *E. coli SMS-3-5* which are inverted with respect to *E. coli APEC O1* are represented as blocks below the central line. The position of Fic protein in *E. coli APEC O1* is shown. This region does not correspond to any homologous region in *E. coli SMS-3-5* which lacks Fic. Probable site of insertion of this region has also been marked on *E. coli SMS-3-5* genome.

Many pathogenic Fic proteins are known to be transferred to their hosts using type IV or type III secretion systems and are encoded by Pathogenicity Island (PAI) 6,10,38. PAIs are family of DNA segments containing virulence gene that have contributed or can contribute to rapid evolution of the virulence capabilities by HGT in various bacterial pathogens. A broader set of genomic entity, called genomic islands (GEIs), contribute not only to pathogenic bacteria but also to non-pathogenic organisms^39^. GEIs often carry integrative and conjugative elements (ICEs) like conjugative transposons, insertion sequences (IS) and integrases to facilitate its lateral transfer and incorporation ‘en bloc'^40^. GEIs are usually inserted in flanking sequences of tRNA genes. Because Fic domains occur both in pathogenic and non-pathogenic organisms and these domains are known to be encoded by PAIs^41^, we hypothesized Fic domains might in general be coded by GEIs. Also, many Fic/Doc proteins have been shown to be part of toxin-antitoxin systems^33^,^42^ which impart stability to GEIs. To test this hypothesis neighborhood of all Fic proteins were analyzed. Interestingly, out of the 21547 unique proteins that we found in 970 Fic neighborhoods 520 were annotated as transposases or IS proteins occurring in 291 Fic neighborhoods and approximately 200 were annotated as integrases occurring in 180 Fic neighborhoods (Fig. 6A). 200 proteins were also annotated as tRNA synthesis related proteins. In order to further confirm the presence of Fic genes on GEIs, predictions of IslandViewer^43^,^44^ were used. IslandViewer uses sequence and genomic neighborhood based approaches to predict GEIs. IslandViewer predicted 343 Fic sequences in 270 genomes to be present on GEIs (pink pars in Fig. 5). Fic/Doc proteins belonging to GEIs are distributed all over the phylogenetic tree (Fig. 5) indicating these proteins have evolved through HGT via GEIs. A closer look at the IslandViewer predictions for *E. coli APEC O1* showed many GEIs, one of them containing the Fic protein (Fig. 6B). GC content of this region is lower than the average GC content of the organism, indicating this gene has been acquired in recent past from an unrelated species via horizontal route. Also, a genome alignment of *E. coli APEC O1* and *E. coli SMS-3-5* revealed the absence of Fic protein containing region in the later genome (Fig. 6C). The alignment clearly shows Fic protein to be inserted between an inverted region (pink and blue boxes). Therefore, *E. coli APEC O1* is as an example where a large chunk of DNA was transferred from unrelated species in recent past i.e., after the divergence of *E. coli SMS-3-5* and APEC O1 species. In few cases after such transfer the organism might lose the mobility genes and become part of stable chromosome. This might be the case for many Fic proteins. Because GEIs contain large chunk of DNA, genes are transferred ‘en bloc’ i.e. genes are transferred along with their neighboring genes. Hence, to confirm the evolution of Fic domains via HGT, domains of neighboring genes were examined for evidences of HGT. Neighboring Fic proteins had 72 unique domains occurring significantly, of which 57 (~79%) showed evidence of HGT in literature (supplementary Figure S4). This further confirms that Fic domains along with its synteny have evolved via HGT. As laterally transferred genes are usually weakly expressed, it has been suggested that horizontal gene transfer occurs farthest from the origin of replication (oriC) or near the terminus^45^. However, as discussed in supplementary results, analysis of chromosomal location of AMPylating enzymes (supplementary Figure S5) did not show any obvious trend indicating their location away from the origin of replication.

### Classification of GS-ATase and study of its evolution

**Figure 7:**
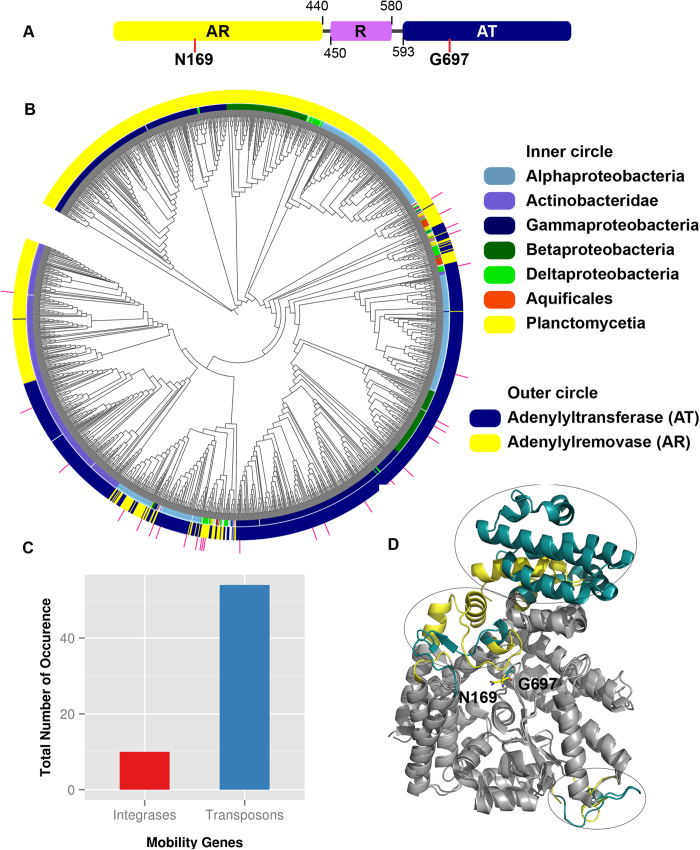
Evolution of AT and AR domains of GS-ATase. (**A**) Depiction of domain boundaries of Adenylylremovase (AR), Regulatory (R) and Adenylyltransferase (AT) domains of GS-ATase. Red lines indicate the structurally equivalent conserved active site residue which change in a class specific manner between AT and AR domains. Other conserved active site residues have not been shown for clarity. (**B**) Phylogenetic analysis of AT and AR domains of GS-ATase. The outer circle is colored based on HMM profile based classification of GS-AT and GS-AR domains, while color coding of inner circle represents the taxonomical distribution of GS-ATase proteins. Pink bars represent GS-ATase domains contained in genomic islands predicted by IslandViewer 43,44. (**C**) Distribution of transposons and integrases in the neighborhood of GS-ATase domain. (**D**) Superposition of 3D structures of AR and AT domains. Structurally similar regions have been colored in grey and dissimilar regions are colored in yellow (AR) and blue (AT). Class specific residue N169 (AR) and G697 (AT) have been represented in sticks.

GS-ATase is a bifunctional enzyme with mutually antagonistic enzymatic activities residing in its two domains which share significant sequence similarity^19^ (Fig. 7). It is indeed intriguing how the opposing enzymatic activities of AMPylation and deAMPylation are catalyzed by domains sharing extensive sequence similarity. We wanted to identify such adenylyltransferase (AT) and adenylylremovase (AR) domains in other organisms to understand their evolution. However, in view of the high homology between AR and AT domains standard BLAST or Pfam domain analysis cannot distinguish these two domains. Therefore, we wanted to identify class specific sequence and structural features of AR and AT domains. Careful structure based analysis of the active site residues of these two domains revealed that, even though most of the active site residues including aspartic acid triad is conserved between AR and AT domains, a crucial difference lie at the position N169. N169 is essential for AR activity but not for AT activity^20^. Hence, the equivalent position in AT is usually occupied by a glycine (Figs. 7A,D). Structural superimposition of AR (PDB ID: 1V4A) and AT (PDB ID: 3NKU) domains also revealed class specific insertions and deletions which might have helped in evolution of AR and AT domains (Fig. 7D, highlighted with circles). Xu *et al.* have proposed that, these conserved indels could be the reason for functional divergence of AR and AT domains^20^. These differences involving conserved indels and conserved class specific residues were used to segregate AR and AT domains. HMMs built using these AR and AT domains were then tested using 5 fold cross validation technique (Supplementary Table 2). Using the HMMs we could distinguish between AR and AT domain with average accuracy and MCC value of 95.79% and 90.85%, respectively. Also, search for AT and AR domains using the HMM profiles developed in the current study revealed that their taxonomical distribution was majorly concentrated in proteobacterial and actinobacterial classes (Fig. 3). AT and AR domain HMMs, developed in the current study, were aligned using HHalign^46^ to search for other class specific residues which could be responsible for distinct clustering on AT and AR domains despite significant similarity in sequence. The HMM-HMM alignment (supplementary Figure S6) revealed that N169 and G697 are the only residues which were conserved in a class-specific manner. However, there were other residues which were conserved in one family but the corresponding positions in the other family were highly variable (supplementary Figure S6). These positions might also be contributing to the separate clustering of AT and AR domains in addition to the N169/G697 pair and class specific indels mentioned earlier.

To gain insight into the underlying determinants of functional divergence in GS-ATase family phylogenetic analysis was performed. Instead of complete sequences of GS-ATase, AT and AR domains, as classified by the HMMs, were taken separately and a phylogenetic tree was built (Fig. 7B). Colors on the inner circle of phylogenetic tree represent taxonomical classes whereas color on the outer circle represent AT/AR domain. AT domains of proteobacterial origin are closer to each other than the AR counterparts of the same genomes and vice versa (Fig. 7). Actinobacteria and a set of alphabacterial sequences are present as sister clades. AT and AR domains of actinobacterial classes segregate separately whereas in case of these alphabacterial sequences there is no segregation of AT and AR domains. It is highly probable that the duplication of AT and AR domain occurred in one of these alphabacterial classes and then was transferred to other proteobacterial and actinobacterial classes via lateral or vertical transfer. GS-ATase from other taxonomical classes like Aquificales, Planctomycetia and Deltaproteobacteria are present as sister clade to alphaproteobacteria and are interwoven indicating these proteins might have evolutionary history of HGT. To confirm this hypothesis we searched for presence of transposons and integrases in the genomic neighborhood of GS-ATase proteins. Interestingly, few proteins from these taxonomical classes had mobility genes in their neighborhood (represented as pink bars on outer circle in Fig. 7B). Figure 7C represents the total count of mobility genes in the genomic neighborhood of GS-ATase. IslandViewer predicted only 10 genomic islands. In contrast to Fic/Doc domains, virulence associated genes, evolution of GS-ATase, a house keeping gene, is not expected to be through HGT, also indicated by our phylogenetic analysis.

### Substrate specificity of DrrA

**Figure 8:**
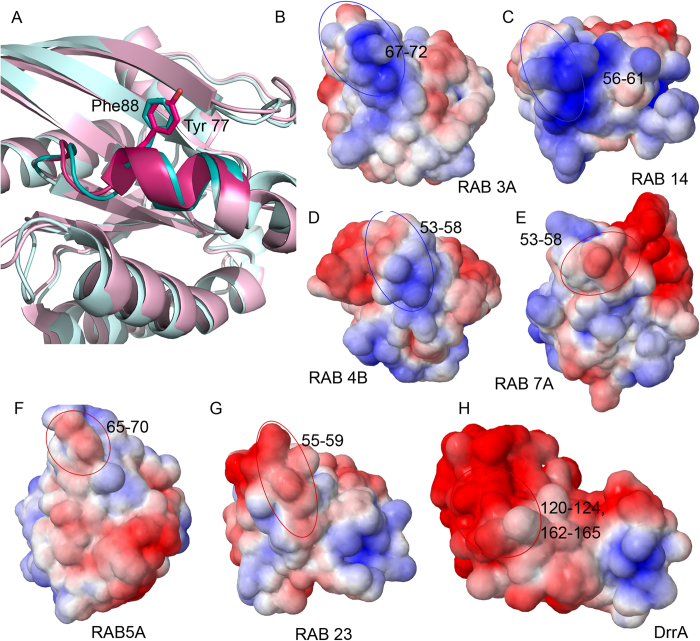
Substrate specificity of DrrA. (**A**) Cartoon representation of Rab1b (PDB ID: 4HLQ, colored pink) and Rab 27a (PDB ID: 3BC1, colored blue). Stick representation depicts Tyr77 of Rab1b, which is AMPylated by DrrA and the structurally equivalent phenylalanine residue in Rab27a. (**B**-**H**) Electrostatic potential (±5 kT/e) rendered onto the surface of different Rab proteins and DrrA, positively charged surface is colored in blue and negatively charged surface in red. The potentials revealed positively charged surface in AMPylation compatible Rab proteins (highlighted in blue circles; B-D) and negative in AMPylation non-compatible Rab proteins (highlighted in red circles; E-G). (**H**) Negatively charged patch mapped on the molecular surface of DrrA.

DrrA is the latest addition to the repertoire of AMPylating enzymes. Though it shares the structural fold of GS-ATase, the sequences do not show significant sequence similarity^5^. DrrA like proteins are very rare and their taxonomical distribution is limited to gammaproteobacterial class, more specifically strains of *Legionella pneumophila* (Fig. 3). Its target specificity has been deciphered and it was shown that DrrA could specifically AMPylate some Rab proteins (Supplementary Figure S7). To understand the substrate preference of DrrA, Rab sequences were analyzed. Eukaryotic Rab proteins can be classified into six sub-groups^47^. Based on the experimental data, broadly it can be said that Group 1, 4, 5 can be AMPylated, while Group 2 and 3 cannot be AMPylated by DrrA. Phylogenetic tree of the above mentioned Rab sequences supported the fact that AMPylation compatible Rabs have some evolutionary features conserved in comparison to AMPylation non-compatible Rabs (Supplementary Figure S7A). Except Rab 6A and Rab 27A AMPylated and non-AMPylated proteins formed separate monophyletic clades. Though Rab27A has features similar to AMPylation compatible Rabs, it lacks the tyrosine which is AMPylated in other Rab proteins and the corresponding residue in Rab27A is a phenylalanine (Fig. 8A). In fact, the switch II region of Rab 27a contains no tyrosine residue. Though Rab6A can be AMPylated it clusters together with non-AMPylated clade. Detailed analysis of the Multiple Sequence Alignment (MSA) of the Rab sequences (Supplementary Figure S7B) revealed sequence attributes contributing to DrrA specificity. The sequence stretch 53 -58 (sequence numbered according to Rab1b from *Homo sapiens*) has an overall positive charge in AMPylated Rab proteins, whereas Rab proteins that cannot be AMPylated by DrrA have a negatively charged or neutral amino acids in this sequence stretch. In the three dimensional structure of Rab proteins, the sequence stretch 53-58 is present on the surface and is in vicinity of Tyr77 which is AMPylated. As can be seen from Fig. 8, the surfaces of Rab proteins which are AMPylated (Fig. 8B–D) and those which cannot be AMPylated (Fig. 8E–G) show distinct differences in electrostatic potentials. Interestingly, two negatively charged stretches in DrrA (120-124, 162-165) come close in three dimensional structure and contribute to a negatively charged patch on the surface of DrrA (Fig. 8H). It is possible that DrrA utilizes this negatively charged surface patch to recognize Rab proteins which have a positively charged region on the surface adjacent to the site of AMPylation. Therefore, our sequence and structural analysis revealed that Rab proteins can be classified as AMPylation compatible and non-compatible based on their surface electrostatic potential which arises from differences in amino acid composition of the sequence stretch 53-58 (human Rab 1b numbering).

## Discussion

AMPylation of proteins has been known since last four decades^2^. The sudden resurgence of interest in this field can be attributed to discovery of AMPylation by two other protein families, namely, Fic and DrrA^5^,^6^, their involvement in host-pathogen interaction, extensive substrate specificity^12^ and their diverse functional roles^31^. In view of the sequence and structural similarity between Fic, Doc and AvrB, they have been grouped together as a single superfamily called Fido. Since Fido and GS-ATase superfamily can potentially catalyze several different types of PTMs, in this work a comprehensive *in silico* analysis involving BLAST, PSI-BLAST, profile HMM and SVMs has been carried out for identifying new Fido and GS-ATase domains from among the unannotated proteins in genomes of various organisms and distinguishing between various functional subfamilies. Systematic benchmarking of different computational protocols revealed that HMMs was distinctly superior to all types of SVM classifiers for identification and classification of various AMPylation subfamilies. Apart from identification of Fic, Doc, AvrB, GS-ATase AT and AR domains, the subfamily specific HMM profiles developed in this work can successfully distinguish AnkX type phosphocholinating domains from Fic type AMPylation domains. The HMM based computational protocol for identification and classification of AMPylating enzymes has also been made available online at http://www.nii.ac.in/novptmenzy.html.

Using the HMM based computational protocol we have identified Fido and GS-ATase superfamily of enzymatic domains from various organisms and classified them into functional subfamilies. Evolutionarily conserved sequence determinants combined with systematic phylogenetic analysis were used to illuminate the functional diversity of Fic/Doc family and understand how protein sequence and function has coevolved. Three putative sub-families that might add to the increasing functional diversity of Fic/Doc family were identified. We expect more biological functions to be unearthed as more number of Fic sequences is experimentally characterized. In a curious evolutionary twist the sequences of bacterial Fic/Doc family was not only interweaved among themselves but also with eukaryotic and archaeal Fic/Doc proteins. The lack of an archaeo-eukaryotic branch also suggested that members of Fic/Doc family might not have been present in last universal common ancestor (LUCA) but have evolved in bacteria and subsequently spread to archaea and eukaryotes via horizontal gene transfer (HGT). We hypothesize that the reason for extensive HGT observed in Fic domains is because it is encoded by highly mobile and unstable GEIs. The evolution of GS-AT and GS-AR domains was studied through an explicitly phylogenetic approach tracing back the duplication and evolution of AT and AR domains. Phylogenetic and genome neighborhood analysis suggested that barring few taxonomical classes GS-ATase have probably evolved through lateral transfer. Phylogenetic analysis of DrrA substrates helped in identification of sequence stretches which are determinants of the substrate specificity of DrrA type AMPylation domains towards a variety of Rab proteins.

## Materials and Methods

### Compilation of dataset

The sequences of all experimentally characterized AMPylating domains belonging to Fic/Doc, AvrB, GS-AT, GS-AR families were compiled based on literature search. This set also included the domains for which crystal structures were available in PDB. They consisted of 12, 1 and 6 sequences from Fic/Doc, AvrB and GS-ATase family respectively. A dataset of protein sequences for each family was compiled using sequence based searches like pair-wise BLASTp 48,49 and PSI-BLAST search against nr database (released in September, 2012) using the experimentally characterized domains as query. BLAST searches were carried out using e-value cut off of 10^-3^. Overlapping hits were obtained in case of BLAST searches for Fic and Doc, because of significant sequence similarity between these two sub-families. In such cases, the obtained hits were classified as Fic or Doc based on their annotation. As AvrB does not share sequence similarity with Fic/Doc family members, no overlapping hits were obtained. Similarly, overlapping hits were obtained for GS-AT and GS-AR domains. In such cases GS-AT and GS-AR domains were classified based on presence of N169 in AR domain and G697 in AT domain^20^. For each class of AMPylating domains redundant sequences sharing very high degree of similarity were removed using BLASTClust program ( ftp://ftp.ncbi.nih.gov/blast/documents/blastclust.html) from the NCBI-BLAST package^48^, so that no two members in a given class shared greater than 60% sequence similarity to each other (Table 1).

### Development of SVM & HMM models for in silico classification of AMPylation domains

SVM^multiclass^ which is based on structural SVMs^50^ was used to develop a machine learning approach for *in silico* identification and correct classification of putative AMPylating domains. SVM^multiclass^ allows classification of multiple classes of data by optimization of the models by varying various parameters like type of kernels (linear, polynomial, radial or sigmoid) and trade-off value (C). For this study kernel was fixed to polynomial whereas C value was varied from 0.001 to 1. The SVMs were trained using features like amino acid composition, dipeptide composition, tripeptide composition and tetrapeptide composition. In addition SVMs were also trained using combination of multiple features, for example amino acid and tripeptide composition together, or amino acid, dipeptide and tetra peptide composition etc. Since the features were composition of Kmers in the sequences of AMPylation domains, the different feature vectors essentially consisted of one dimensional arrays of size 20^k^ and the i^th^ element of the array is fraction of k-mer of type i.


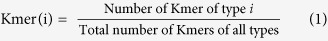


where i = 1, 20^K^. Thus the size of the feature vector corresponding to K = 1 i.e. amino acid composition is 20, while size of feature vectors for di-, tri- and tetra-peptide composition are 400, 8000 and 160000 respectively. Similarly these feature vectors were combined suitably to obtain feature vectors corresponding to multiple k-mer composition. SVM models for each family of AMPylation domains were developed by using sequences belonging to the given family as positive dataset and sequences belonging to the other families as negative dataset.

HMMs are statistical models that capture the consensus information from a set of related protein sequences at various sequence position. In order to develop profile HMM models for classification of AMPylation domains HMMER3^51^ was used. Unlike SVM models HMM profiles for each family were developed using only the positive datasets. HMM for each family was built using multiple sequence alignment (MSA) of non redundant set of proteins These HMM models are available online at http://www.nii.ac.in/novptmenzy.html.

### Evaluation of performance of SVM and HMM models

The performance of all the SVMs as well as HMMs was evaluated using fivefold cross validation methods. For 5-fold cross validation the total data set was randomly divided into five equal sized sets. One of these five datasets was used as test set while the remaining four sets were used to train the SVM models or derive the profile HMMs. This process was repeated five times such that each of the five parts was used for training as well as testing. The trade-off parameter (C) for the SVM^multiclass^ was optimized based on the results of the 5-fold cross validation tests. Sensitivity (SN), specificity (SP), accuracy (ACC), Mathew’s Correlation Coefficient (MCC), F1 and normalized percentage better than random (S) were used as statistical measures to evaluate the performance of cross validation test. For Fic/Doc family both SVM and HMM were developed and performances of both the methods in identification and classification of Fic, Doc and AvrB were compared using the test dataset. Since the performance of HMM method was found to be distinctly superior to that of SVM, for analysis of AnkX, AR, AT and DrrA family members only HMM profiles were developed. Similarly for subsequent phylogenetic analysis of Fic, Doc, AR, AT and DrrA families sequences were identified from various genomes by using HHM profiles only.

### HMM models for identification of AnkX proteins

The Fic domain of AnkX shares a high sequence similarity with other Fic domains. Hence, HMMs based on its Fic domain could not be used to distinguish AnkX type Fic domains. Since AnkX proteins also contain a CMP binding domain (1-46, 291-328) and a unique insert within the Fic domain (110-180) (Fig. 1), these sequence stretches were used to build HMM profiles for identifying AnkX type proteins. A BLAST search using AnkX protein sequence gave 23 hits with an e-value cut off of 0.001. Highly similar sequences sharing more than 95% sequence similarity were removed. These sequence stretches corresponding to the CMP-binding domain and insert domains were extracted from a non-redundant set of 7 AnkX homologs and aligned using ClustalW2. Based on these alignments HMM profiles for AnkX was built using HMMER3. The HMM was used to search NCBI nr database. The e-value cut off used for this HMM search was 1. D28, R30, Y41 and R44 have been shown to be important in CMP binding in AnkX. These conserved residues were used to crosscheck the HMM results. If 3 out of 4 residues were conserved and a Fic domain was present it was considered as a positive hit.

### Analysis of phylogeny and synteny of AMPylating enzymes

Fic/Doc sequences were obtained by searching in nr database using the Fic and Doc HMMs developed in this study. Sequences were classified into Fic or Doc subfamilies based on the e-value for the HMM profile match. Redundant sequences were removed by clustering them at a percentage similarity cut off of 60% using BLASTClust. In order to build the phylogenetic tree the sequences were aligned using ClustalW2 and bootstrapped trees (1000 replicates) were built using Quick Tree tool of Phylip package^52^. The iTOL^53^ utility was used for visualization and analysis of the phylogenetic trees. Profiles were built using HHPred^54^ for sequences of known Fic structures and Fic motif was extracted by aligning the Fic/Doc sequences to this profile. Consensus motif of sequences in each clade was also calculated using Skylign^55^ and motif positions 1 to 9 and 12 were used to build the HMM logo. The leaves of the tree were colored based on the taxonomical classes or presence of different Fic/Doc motifs. For visualization of the active site motifs on the three dimensional structures of Fic/Doc domains, one representative member was chosen from each group. Doc protein from *Enterobacteria phage P1* was the representative structure from Doc class (colored red in Fig. 4 ) and the active site was mapped on the available crystal structure (PDB ID: 3K33). Similarly, crystal structure of HpFic from *Helicobacter pylori* (PDB ID: 2F6S) was used to visualize active site of the Fic class (colored blue in Fig. 4). For the remaining three classes representative sequences were chosen and their 3D structures were modeled based on homology using SWISS-MODEL^56^,^57^. The NCBI accession number for the representative sequences for the groups colored blue, green and yellow are 120555568, 299469428 and 319943128 respectively.

Genomic neighborhoods of AMPylation domains identified in different organisms by our profile based search were analyzed if the fully/partially sequenced genomes were available. Such genomic neighborhood analysis could be carried out for AMPylation domains from 970 organisms. Pfam domain information resource was utilized to find out domain annotations for five upstream and five downstream neighbors of AMPylation domain containing genes. Pfam domains which were found in the neighborhood of AMPylation domains at least 40 times were further analyzed to find out literature based evidence about these neighboring domains having evolutionary history of Horizontal Gene Transfer (HGT). Cytoscape^58^,^59^ software was used to represent the Pfam domain for the neighboring genes as nodes and the size of each node denoted number of occurrences of genes in Fic neighborhood.

In order to analyze the transposons and integrases in the genomic neighborhood of AMPylation domains, neighboring genes annotated as transposon, transposase or insertion sequences were categorized under a broad heading of transposons and genes annotated as integrases were counted separately. In addition Genomic Island (GEI) predictions were carried out by using the IslandViewer^43^,^44^ software to decipher the role of HGT in evolution of AMPylating enzymes. IslandViewer is a web-based application that combines different sequence based and genomic neighborhood based approaches like IslandPick, IslandPath-DIMOB and SIGI-HMM, for prediction of GEIs. IslandPick predicts horizontally transferred genes based on analysis of genomic neighborhoods in related strains^60^. IslandPath-DIMOB uses atypical sequence composition such as dinucleotide composition bias and presence of mobility genes like transposon, insertion sequence and integrases for prediction of GEIs^61^. SIGI-HMM uses HMM to analyze codon usage of a gene to identify potential GEIs^62^. IslandViewer combines these different prediction methods and identifies GEIs in a given genome. Since pre-computed results of IslandViewer were available for all published prokaryotic genomes, we utilized those predictions to identify GEIs in the neighborhood of genes harboring AMPylating domains. For detailed visualization of HGT, Mauve^63^,^64^ whole genome alignment tool was used.

### Substrate specificity of DrrA

Since substrate specificity of DrrA towards a number of Rab proteins were known, phylogeny of known DrrA substrates were also analyzed. Protein sequences of Rab 1a, 1b, 35, 8a, 13/8c, 3a, 37/26b, 27a, 5a, 22a, 31, 7a, 9a, 23, 32/32a, 38/32b, 4b, 11a, 14 and 6a were downloaded from Rab database ( http://bioinformatics.mpibpc.mpg.de/rab/)^47^. Sequences were aligned using ClustalW^65^ and the alignment was visualized using JalView^66^. Bootstrapped phylogenetic tree was built as mentioned earlier. Substrate preference of DrrA was marked on to the tree using iTOL software. Three dimesional structures of AMPylating enzyme DrrA, AMPylation compatible substrates Rab 3a (PDB ID: 3RAB), Rab 14 (PDB ID: 4DRZ), Rab 4b (PDB ID: 2O52) and AMPylation incompatible substrates Rab7a (PDB ID: 3LAW), Rab 5a (PDB ID: 3MJH), Rab 23 (PDB ID: 1Z22) were used to compute the electrostatic surfaces of DrrA and its potential substrates to understand specificity of recognition. The APBS/PDB2PQR website ( http://nbcr-222.ucsd.edu/pdb2pqr_2.0.0/)^67^,^68^ was used to calculate and visualize the electrostatic potentials.

## Additional Information

**How to cite this article**: Khater, S. and Mohanty, D. *In silico* identification of AMPylating enzymes and study of their divergent evolution. *Sci. Rep.* **5**, 10804; doi: 10.1038/srep10804 (2015).

## Supplementary Material

Supplementary Information

Supplementary Dataset 1

## Figures and Tables

**Table 1:** Number of sequences belonging to each sub-family.

**AMPylation subfamilies**	**No. of sequences used to build profiles**	**No. of sequences from nr search using profiles**	**No. of sequences used for phylogenetic analysis**
Fic	452	3614	1346^a^
Doc	99	1466	531^a^
AvrB	9	21	—
AnkX	7	27	—
AT of GS-ATase	89	1356	831^b^
AR of GS-ATase	198	1369	759^b^

^a^Sequences at 60% redundancy.

^b^Sequences at 95% redundancy

^*^NCBI accession numbers corresponding to each set of sequences mentioned above are available in Supplementary File 2
