# Supplementary material for: In silico identification of AMPylating enzymes and study of their divergent evolution
Source: Sci Rep. 2015 Jun 3;5:10804. doi: 10.1038/srep10804 (PMC4454073; doi:10.1038/srep10804)
Supplement: Supplementary Information [file srep10804-s1.pdf]

Supplementary Data

In silico identification of AMPylating enzymes and study of their divergent evolution

Shradha Khater and Debasisa Mohanty[§]

Bioinformatics Center, National Institute of Immunology, Aruna Asaf Ali Marg,
New Delhi – 110067, India.

[§]Corresponding author: Debasisa Mohanty

E-mail: deb@nii.res.in

Supplementary Methods

Statistical measures for evaluation of performance by SVM and HMM

The values for SN, SP, ACC and MCC were computed using the standard formulae as:

$$\text{Sensitivity} = \frac{\text{TP}}{\text{TP} + \text{FN}} * 100$$

$$\text{Specificity} = \frac{\text{FP}}{\text{FP} + \text{TN}} * 100$$

$$\text{Accuracy} = \frac{\text{TP} + \text{TN}}{\text{TP} + \text{FN} + \text{FP} + \text{TN}} * 100$$

$$\text{MCC} = \frac{(\text{TP} * \text{TN}) - (\text{FP} * \text{FN})}{\sqrt{(\text{TP} + \text{FN}) * (\text{TN} + \text{FP}) * (\text{TP} + \text{FP}) * (\text{TN} + \text{FN})}} * 100$$

where, for a given family True Positive (TP) is number of sequences which were correctly predicted family members; False Positive (FP) is number of sequences belonging to other families which were wrongly predicted as family members; True Negative (TN) is number of sequences belonging to other families which were correctly predicted as non-family members and False Negative (FN) is number of sequences belonging to the given family which were wrongly predicted as non-family members.

Apart from the above mentioned performance measures, it was also tested whether the predictions made by the classifiers were significantly better than random classifications. For this the number of sequences expected to be predicted correctly by a random predictor, R we first calculated using the formulae [1]:

$$\text{R} = \frac{(\text{TP} + \text{FN}) * (\text{TP} + \text{FP}) + (\text{TN} + \text{FN}) * (\text{TN} + \text{FP})}{\text{TP} + \text{FN} + \text{FP} + \text{TN}}$$

Then the performance of the classifier compared to random predictions i.e. normalized percentage better than random (S) was evaluated as:

$$\text{S} = \frac{(\text{TP} + \text{TN}) - \text{R}}{\text{TP} + \text{FN} + \text{FP} + \text{TN} - \text{R}} * 100$$

The measure S is independent of total sample size and a score of S=0% represents totally random classifier whereas a score of S=100% represents perfect classifier.

Predictions were also benchmarked using another robust statistical measure F1 which is the trade-off between precision and recall. F1 measure is the harmonic mean between precision and recall and was computed using the formulae:

$$\mathrm{F1} = \frac{2 * \mathrm{TP}}{2 * \mathrm{TP} + \mathrm{FN} + \mathrm{FP} + \mathrm{TN}} * 100$$

Based on the above measures optimum SVM and HMM were chosen for *in silico* classification of AMPylation domains.

Principal Component Analysis

Principal Component Analysis (PCA) is a statistical procedure that reduces the dimensionality of high-dimensional data while capturing largest part of variance in the data. PCA analysis was used on various features of SVM to find which feature provides maximum discriminative power to SVMs. Principal Components (PCs) were calculated using stats::prcomp function of R package and the scatter plots were visualized using ggplot2 of R package [2].

Chromosome mapping of Fic/Doc and GS-ATase gene products

In order to know on which part of the chromosome, with respect to the origin of replication (oriC), genes for AMPylating enzymes are located, we downloaded the DoriC database[3] which contains predicted oriC regions of approximately 2700 bacterial genomes and 100 archaeal genomes. The database gives the information like accession number of genomes, start and stop positions of oriC and length of genome. Search for Fic, Doc and GS-ATase sequences in these 2700 bacterial genomes using the developed HMMs yielded 484 Fic sequences (from 310 genomes), 184 Doc sequences (from 167 genomes) and 632 GS-ATase sequences (from 546 genomes) were also obtained**.** The region coding these genes (start and stop positions) were obtained by querying the NCBI database using in-house Perl script. To calculate the distance between these genes and oriC mid-points or center-points of genes and oriC was considered. Hence, the absolute distance between genes and oriC (D) can be calculated as:

$$D = |\mathrm{G} - \mathrm{C}|$$

where, G is the center point of gene and C is the center point of oriC.

As the distance from oriC of different AMPylating enzymes needs to be compared, therefore the obtained distance, D, was normalized and normalized distance(ND) was calculated.

If D ≤L/2,

$$\mathrm{ND} = \frac{\mathrm{D}}{\mathrm{L}} * 100$$

Else,

$$\mathrm{ND} = \frac{\mathrm{L} - \mathrm{D}}{\mathrm{L}} * 100$$

where, L is the length of genome. An ND value of 50 would indicate genes farthest from oriC , i.e. near the termination site, and an ND of 0 would indicate genes closest to oriC.

Normalized distance was calculated for 484 Fic sequences, 184 Doc sequences and 632 GS-ATase sequences. For each family of enzymes percentage population in the normalized distance bracket of 0-5, 5-10, 10-15 and so on was calculated and represented as a plot using ggplot[2] package of R.

Supplementary Results

Horizontal gene transfer in eukaryotes

Eukaryotic Fic proteins have been transferred from various bacterial sources via different HGT event. Some of the eukaryotic Fic proteins belonging to *Basidiomycota* division of fungi (E1) are sister to proteins from cyanobacterial species. Interestingly, *Basidiomycota* and cyanobacteria are known to be the main components of lichens. Though HGT between components of symbionts is rarely seen[4], sharing of same niche disposes them to be recipients of related gene groups by same or similar bacterial donors. Therefore, we hypothesize that two separate events of HGT from a prokaryotic donor to cynobacterial and *Basidiomycota* might have occurred. In E2 clade a group of amoeba (*Dictyostelium* species), plant and fungal Fic proteins are clubbed together with proteins of bacterial origin. Gene transfer from bacteria into social amoeba *Dictyostelium discoidium* has been described earlier[5] based on the “you are what you eat” hypothesis[6]. The endosymbiotic gene transfer would have occurred in *Dictyostelium* or its ancestor and then was

vertically transferred to fungal and plant species. Fic/Doc proteins from metazoan classes of eukaryotes like ant, sea squirts and humans (E3) show an evidence of evolution through a separate prokaryote-to-eukaryote HGT event. Some fungal proteins (E4) show evidence of Doc-like proteins being laterally transferred from bacterial donors via a discrete HGT event.

Chromosome mapping of Fic/Doc and GS-ATase gene products

As laterally transferred genes are usually weakly expressed, it has been suggested that horizontal gene transfer occurs farthest from the origin of replication (oriC) or near the terminus[7]. Fic and Doc enzymes, which show evidences of horizontal gene transfer, should cluster furthest from the oriC. Therefore, we performed chromosome mapping of AMPylating enzymes. Normalized distance (Supplementary method) from oriC was calculated for AMPylating enzymes. The distribution (**Figure S5**) of the normalized distance from OriC for the genes encoding these ethree AMPylating enzyme (Fic, Doc and GS-ATase), did not show any trend of clustering near the oriC or near the terminus. It may be noted that in an earlier study on organization of bacterial genomes[7], even though Rocha *et al* did observe underrepresentation of HGT elements in the vicinity of oriC, they failed to observe any tendency of HGT elements to cluster near the terminus.

SUPPLEMENTARY TABLES

Supplementary Table 1: Fivefold cross validation results for various classifiers of Fic/Doc and AvrB family

	C	Family	Sensitivity	Specificity	Accuracy	MCC	F1	%S
Amino acid composition	0.001	Fic	93.58	36.11	82.50	35.87	89.62	100.22
	0.001	Doc	33.33	94.36	83.57	34.41	41.77	32.91
	0.001	AvrB	66.67	99.46	98.93	66.12	66.67	66.12
		**Avg**	**64.53**	**76.64**	**88.33**	**45.47**	**66.02**	**66.42**
Dipeptide composition	0.1	Fic	97.57	29.63	84.46	40.30	91.02	100.19
	0.1	Doc	30.30	97.61	85.71	40.88	42.86	36.26
	0.1	AvrB	22.22	100.00	98.75	46.84	36.36	35.99
		**Avg**	**50.03**	**75.75**	**89.64**	**42.68**	**56.75**	**57.48**
Tripeptide composition	0.001	Fic	98.89	12.04	82.14	24.45	89.94	100.21
	0.001	Doc	11.11	98.92	83.39	22.96	19.13	14.95
	0.001	AvrB	22.22	100.00	98.75	46.84	36.36	35.99
		**Avg**	**44.08**	**70.32**	**88.10**	**31.42**	**48.48**	**50.38**
Tetrapeptide composition	0.01	Fic	95.13	57.41	87.86	58.05	92.67	100.16
	0.01	Doc	57.58	95.23	88.57	57.87	64.04	57.35
	0.01	AvrB	55.56	100.00	99.29	74.27	71.43	71.10
		**Avg**	**69.42**	**84.21**	**91.90**	**63.40**	**76.05**	**76.20**
Amino acid + Dipeptide composition	0.001	Fic	93.36	58.33	86.61	54.81	91.84	100.18
	0.001	Doc	56.57	94.14	87.50	54.44	61.54	54.14
	0.001	AvrB	77.78	99.46	99.11	73.34	73.68	73.23
		**Avg**	**75.90**	**83.98**	**91.07**	**60.86**	**75.69**	**75.85**
Amino acid + Tetrapeptide composition	0.01	Fic	94.91	44.44	85.18	46.67	91.18	100.19
	0.01	Doc	42.42	95.66	86.25	46.30	52.17	44.64
	0.01	AvrB	66.67	99.46	98.93	66.12	66.67	66.12
		**Avg**	**68.00**	**79.85**	**90.12**	**53.03**	**70.01**	**70.32**
Amino acid +Dipeptide +Tetrapeptide composition	0.001	Fic	95.35	50.93	86.79	53.31	92.09	100.17
	0.001	Doc	48.48	95.66	87.32	51.56	57.49	50.33
	0.001	AvrB	77.78	99.82	99.46	82.23	82.35	82.08
		**Avg**	**73.87**	**82.14**	**91.19**	**62.37**	**77.31**	**77.53**
HMM		Fic	81.20	97.27	84.29	65.57	89.26	61.03
		Doc	88.89	94.14	93.22	78.90	82.54	78.38
		AvrB	100.00	100.00	100.00	100.00	100.00	100.00
		**Avg**	**90.03**	**97.14**	**92.50**	**81.49**	**90.60**	**79.80**

Supplementary Table 2: Fivefold Cross Validation results for various classifiers for AR and AT domains of GSATase.

	Family	Sensitivity	Specificity	Accuracy	Matthews Correlation coefficient	F1	%S
Averages	AT	94.45	98.89	95.79	90.85	96.87	90.46
	AR	98.89	94.45	95.79	90.85	93.57	90.46
	**Avg**	**96.67**	**96.67**	**95.79**	**90.85**	**95.22**	**90.46**

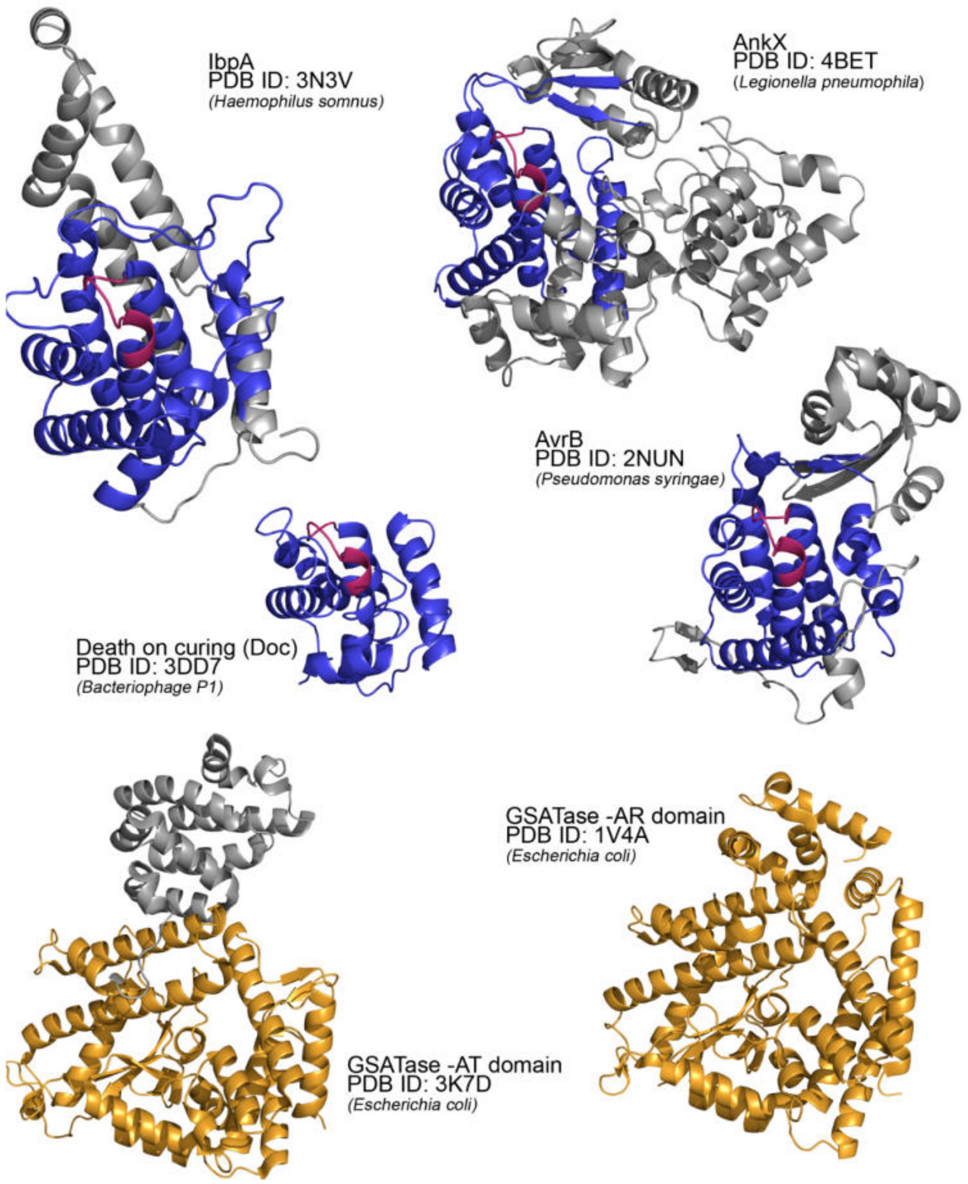

Supplementary Figure S1: Fido (blue) and GSATase (orange) fold (B) Though Fido and GSATase fold proteins usually catalyze AMPylation they share no fold level similarity. The Fido fold itself is highly diverged, with proteins containing additional helices, insertions and deletions.

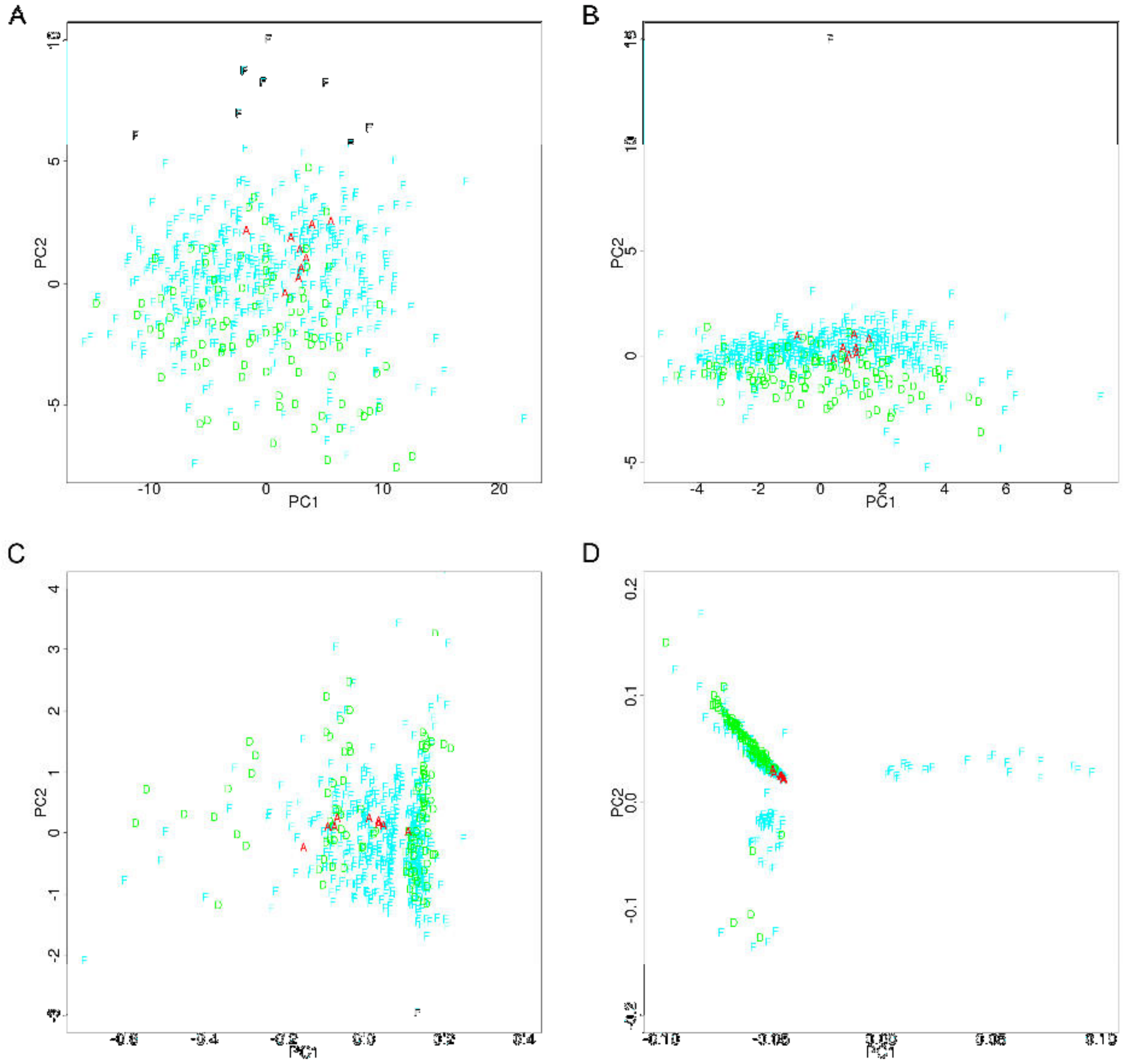

Supplementary Figure S2: PCA analysis of various SVM classifiers (A) Amino acid composition, (B) dipeptide composition, (C) tripeptide composition and (D) tetrapeptide composition. Principal Component Analysis (PCA) was done in feature vector space to understand which classifier can separate Fic, Doc and AvrB sequences to maximum extent. The top two principal components have been plotted as scatter plot. Each point is labeled by an alphabet corresponding to initials of Fic, Doc and AvrB. Fic sequences are represented in blue color, Doc in green and AvrB in red.

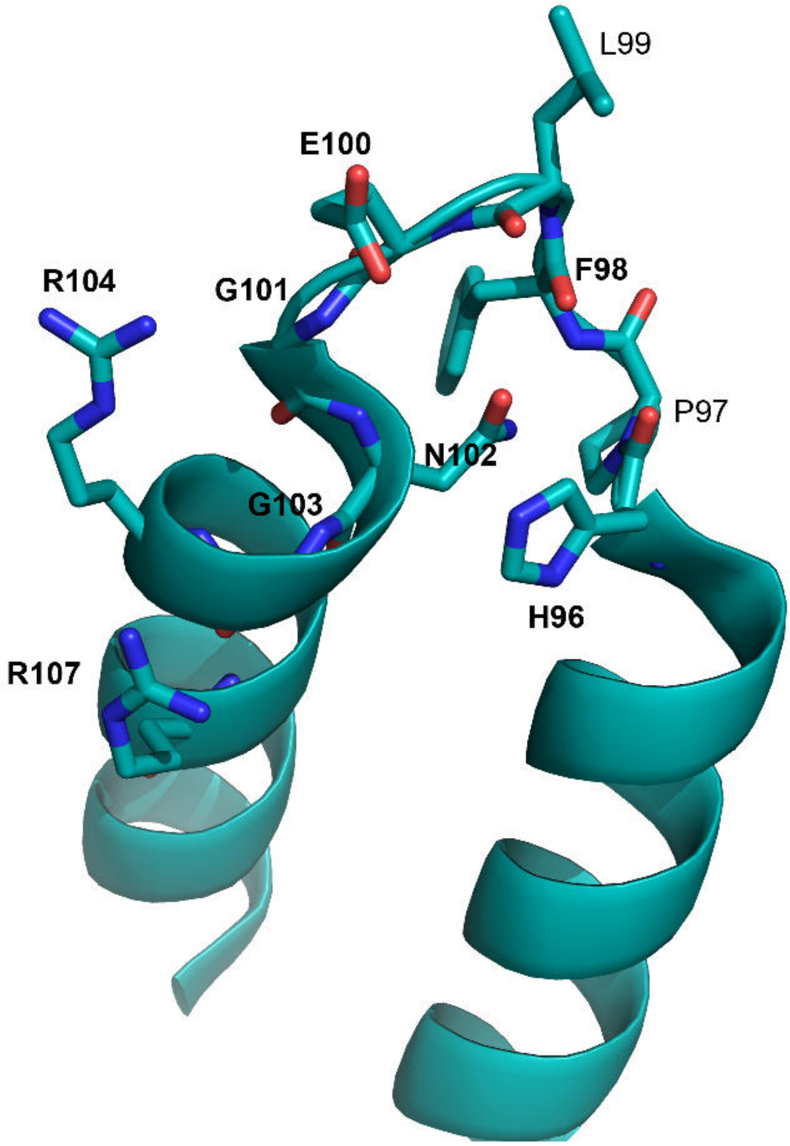

Supplementary Figure S3: The three dimensional structure (residues 96-107 in 2F6S) of the sequence stretch HPFLEGNGRATR in HpFic (Fic domain from *Helicobacter pylori*) corresponding to the conserved sequence motifs shown in **Figure 4**. The active site residues are shown in bold font.

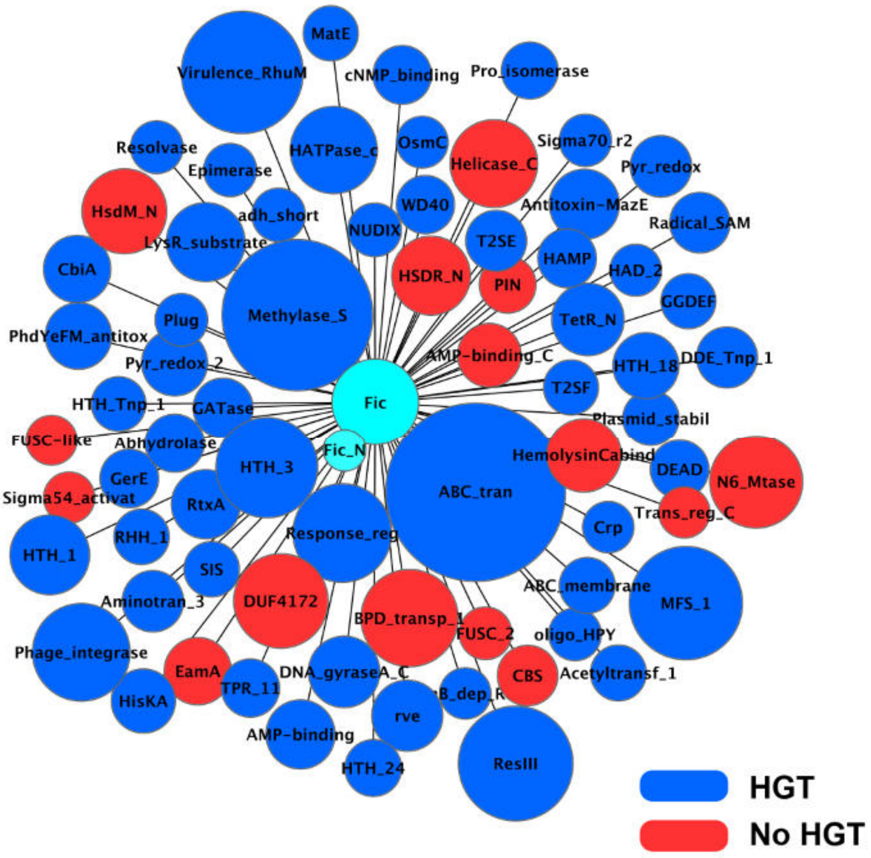

Supplementary Figure S4: Proteins in genomic neighborhood of Fic/Doc proteins. Occurrence of proteins containing different Pfam domains in the synteny of Fic/Doc proteins depicted as a graph having Pfam domains as nodes. An edge between Fic/Doc domain and a given Pfam domain indicate occurrence of the corresponding Pfam domain in the genomic neighborhood of Fic/Doc proteins. The size of nodes represents the frequency of occurrence of the corresponding domains. Blue and red color on node represents literature based evidence for evolution of the corresponding Pfam domain through horizontal gene transfer (HGT).

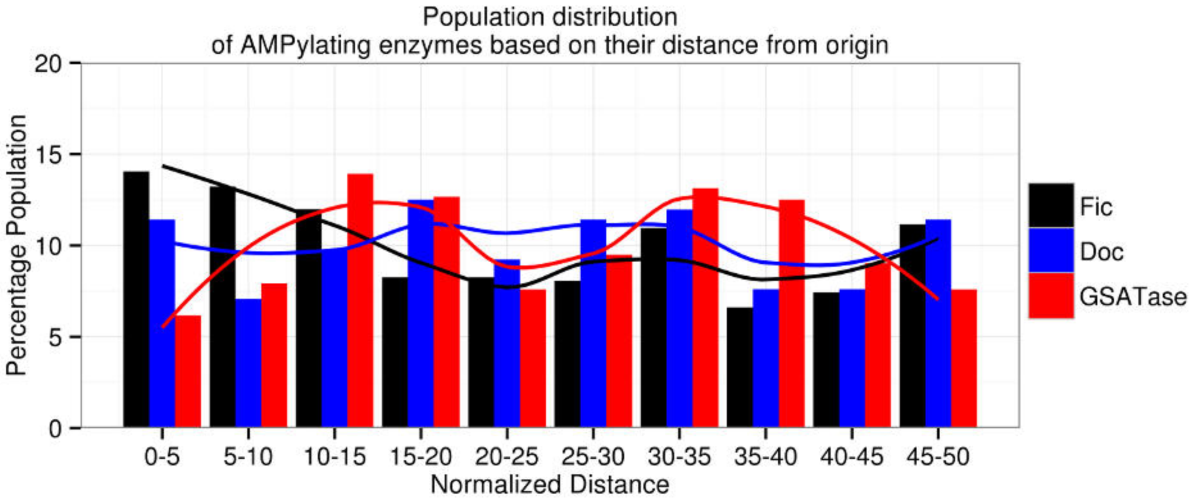

Supplementary Figure S5: Chromosome location of AMPylating enzymes. The plot represents population distribution of AMPylating enzymes based on their normalized distance from origin of replication (oriC).

```
 Query         313673821_17_293

 No Hit                              Prob E-value P-value  Score    SS Cols Query HMM  Template HMM
  1 114776239_503_779               100.0 3.6E-67 3.6E-67  448.3   0.0  245    1-271   17-263 (276)

No 1
>114776239_503_779
Probab=100.00  E-value=3.6e-67  Score=448.29  Aligned_cols=245  Identities=22%  Similarity=0.270  Sum_probs=208.5

Q ss_pred                ChHHHhhhCHHHHHHHHHChHHHHHHhcchhhhhhhh-HHHHHHHHhhhccccccccccccCHHHHHHHHHHHHHHHHHH
Q 313673821_17_2    1 LLTPVLKHSIFLKNKIFTEPSLAYSAFNKLNSIRDKVT-IQSELLNPSNIEVNNQNSEIFEMPETKFLEYLRDFKYIEYI   79 (276)
Q Consensus         1 ~L~~l~~~S~~l~~~l~r~P~~l~~l~~~~~~~~~~~~~~~~~~~~~~~~~~~~~~~~~~~~~~~~~~~LR~~r~~~~l   79 (276)
                        .|+.+++.|+|+++.|.+||+.++++++.......... ...+.+..            ...|.++.+++||+||+++++
T Consensus        17 ~L~~ll~~S~~l~~~L~r~P~~ld~Ll~~~~~~~~~~~~~~~l~~~l--------------~~~d~e~~~~~lR~~k~~e~l   84 (276)
T 114776239_503_  17 WLTGVLSASRYLADHIVKDPSWLEWPLMVEHSDADIRDLCTQLNQLS------------GFDDVEQVLADIGRGVDRARL   84 (276)
T ss_pred                HHHHHHccCHHHHHHHHHChHHHHHhcCcccccccCcchhHHHHHHHH------------hcCCHHHHHHHHHHHHHHHHHH
Confidence          4789999999999999999999999888743222111 11111111            11156889999999999999

Q ss_pred                HHHHHHhcCCCCHHHHHHHHHHHHHHHHHHHHHHHHHHHHHHHhccCCCCCcCCCCCeEEEeccccCCccccccccceEEE
Q 313673821_17_2   80 IIALEELYFKKDILEITAHISAFASAMLEISYRYAYRFLEEKYGKPKDEEDRDVPFSVIGLGKLGGWELNFSSDIDIMYV  159 (276)
Q Consensus        80 rIa~~Dl~g~~~l~~v~~~LS~lAda~l~~al~~~~~~~~~~~~~~~~~~~~~~~~~~~VigMGKLGg~ELNysSDIDLIfv  159 (276)
                        ||+++|+.|..++.+|..+||+|||++|+++++.++.++.            .|..   .+|+||||||||+||-|.||+|||||
T Consensus        85 Ria~~dl~g~~~~~~vs~~Lt~lAeavl~~al~~a~~~l~-------~P~~~~~~~faVIg~GklGG~ElGY~SDlDlvFv  155 (276)
T 114776239_503_  85 LSALAVDAHTADAMTIGGWLADIADAATQAVLRLCLHEMG------LPAD---FPFVALAMGKHGSREMGMVSDLDMVFV  155 (276)
T ss_pred                HHHHHHHhCCCCHHHHHHHHHHHHHHHHHHHHHHHHHHHHcc------CCCC---CCEEEEeccCcCCccCcccceEEEE
Confidence          99999999999999999999999999999999998776       2320   2799999999999999999999999

Q ss_pred                ecCc-cccccccccccchHHHHHHHHHHHHHHHHHhcCCCCceEEeeCCCCCCCCCCccccCHHHHHHHHHHHhhHHHHH
Q 313673821_17_2  160 YGTE-KGKTTGGSKGRLSNHEFFVKLGEKIKYYLNERTEKGFVYRVDLRLRPDGDRGPLALPIRSYETYYELYGQSWERM  238 (276)
Q Consensus       160 y~~~~~~~~~~~~~~~~~~~~f~rl~~~li~~L~~~T~~G~v~rVDlRLRP~G~~GpLv~Sl~a~e~YY~~~gr~WER~  238 (276)
                        |++. ++...+  .++++  +||.+|+|++++.|++.|..|.+|+||+||||+|++||||.|+++|++||.+|+++||+|
T Consensus       156 ~~~~~~~~~~~g~~~~~~~~~~~~rL~q~l~~~l~~~~~~g~lyevD~rLRP~G~sG~Lv~sl~af~~Y~~~~Aw~WE~Q  231 (276)
T 114776239_503_ 156 LVHDDPSQMLG--RRSVG--EHGQRIGRRMIQYLTGKPPFGAGFEFDARLRPSGSSGVLVTSITGFRAYQFHDAQTWEHQ  231 (276)
T ss_pred                eeCCCccccccc--chhHH--HHHHHHHHHHHHHHhcccCCCCCceeeeecCcCCCCCCCCCceecHHHHHHHHhhhhhhHHHH
Confidence          9887 542222  35666  99999999999999999999999999999999999999999999999999999999999

Q ss_pred                HHHhccccCCHHHHHHHHHHhccCceEeccCch
Q 313673821_17_2  239 MLLKGTVVAGDEEFGERLLKNLRPFIFRRSIDY  271 (276)
Q Consensus       239 AliKAR~vAGd~~~~~~~~~~~~~~~~pfv~rr~ld~  271 (276)
                        ||+|||||||||.+++..+... +|.+|.+.=|.
T Consensus       232 AL~RAR~vAGd~~l~~~~~~~~~~~r~~~l~~~r~~  263 (276)
T 114776239_503_ 232 ALCRARAVAGPIAACAAVEEV-VSSVLSQPRDA  263 (276)
T ss_pred                HHHhhcccCCCHHHHHHHHHHh-hhhhcCCCCCc
Confidence          9999999999998888774 88888866444
```

Supplementary Figure S6: Alignment of HMM profiles. HMM profiles of AT and AR domains of GS-ATase were aligned using HHalign software[8]. The red colored box indicate residue which is conserved in a class specific manner. The consensus sequence and secondary structures have also been depicted in the alignment.

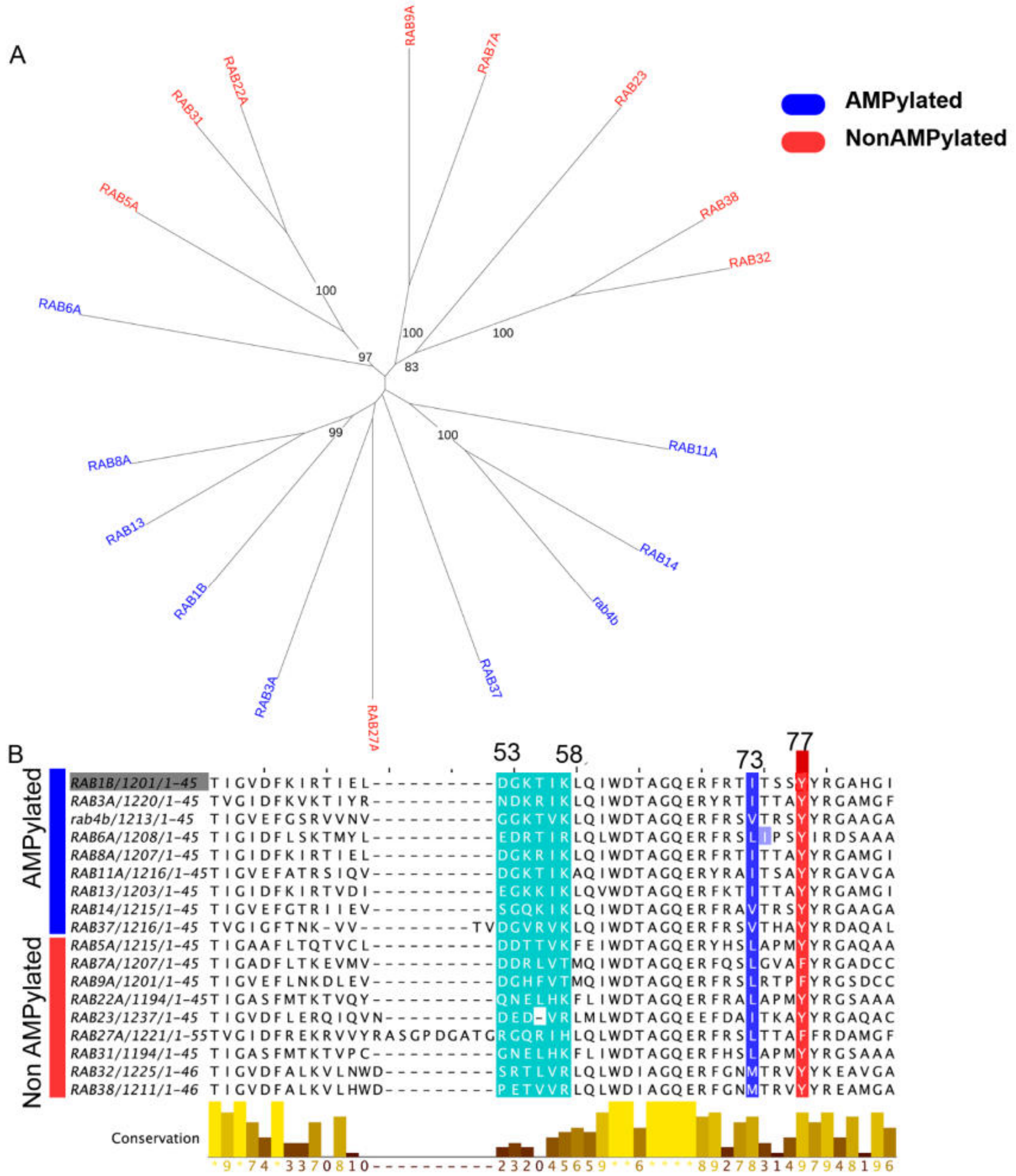

Supplementary Figure S7: Substrate specificity of DrrA (A) Phylogenetic tree of Rab proteins with leaves colored based on whether the corresponding Rab protein is AMPylated (Blue) by DrrA or not AMPylated (red). (B) Multiple sequence alignment (MSA) of switch 1 and switch 2 regions of Rab proteins. The column depicted in red color represents the tyrosine which is AMPylated in Rabs. substrates of DrrA. Cyan represents residue stretch 53 – 58 which has more number of positively charged residues in Rabs which are AMPylated by DrrA compared to the Rab proteins which are not AMPylated. AMPylation compatible and non-compatible Rab proteins have been marked in blue and red, respectively.

REFERENCES

1 Shepherd, A. J., Gorse, D. & Thornton, J. M. Prediction of the location and type of beta-turns in proteins using neural networks. *Protein science : a publication of the Protein Society* **8**, 1045-1055, doi:10.1110/ps.8.5.1045 (1999).

2 Wickham, H. *ggplot2: elegant graphics for data analysis*. (Springer New York, 2009).

3 Gao, F., Luo, H. & Zhang, C. T. DoriC 5.0: an updated database of oriC regions in both bacterial and archaeal genomes. *Nucleic Acids Res* **41**, D90-93, doi:10.1093/nar/gks990 (2013).

4 Dal Grande, F., Widmer, I., Wagner, H. H. & Scheidegger, C. Vertical and horizontal photobiont transmission within populations of a lichen symbiosis. *Molecular ecology* **21**, 3159-3172, doi:10.1111/j.1365-294X.2012.05482.x (2012).

5 Eichinger, L. *et al.* The genome of the social amoeba Dictyostelium discoideum. *Nature* **435**, 43-57, doi:10.1038/nature03481 (2005).

6 Doolittle, R. F., Feng, D. F., Anderson, K. L. & Alberro, M. R. A naturally occurring horizontal gene transfer from a eukaryote to a prokaryote. *Journal of molecular evolution* **31**, 383-388 (1990).

7 Rocha, E. P. The replication-related organization of bacterial genomes. *Microbiology* **150**, 1609-1627, doi:10.1099/mic.0.26974-0 (2004).

8 Soding, J. Protein homology detection by HMM-HMM comparison. *Bioinformatics* **21**, 951-960, doi:10.1093/bioinformatics/bti125 (2005).
